# Fludarabine attenuates inflammation and dysregulated autophagy in alveolar macrophages via inhibition of STAT1/IRF1 pathway

**DOI:** 10.1186/s42826-025-00245-7

**Published:** 2025-05-07

**Authors:** Jooyeon Lee, Jeong-Ran Park, Hanbyeol Lee, Seok-Ho Hong, Woo Jin Kim, Oliver Eickelberg, Sung-Min Park, Semin Ryu, Sung Joon Cho, Seung-Jin Kim, Se-Ran Yang

**Affiliations:** 1 Department of Thoracic and Cardiovascular Surgery, Kangwon National University, Chuncheon, Gangwon, South Korea; 2 Department of Internal Medicine, Kangwon National University, Chuncheon, Gangwon, South Korea; 3 Division of Pulmonary and Critical Care Medicine, Department of Medicine, University of Pittsburgh, Pittsburgh, PA USA; 4 Institute of Medical Science, School of Medicine, Kangwon National University, Gangwon State, 1 Kanwondaehak-Gil, Chuncheon, 24341 South Korea; 5 Department of Biochemistry, College of Natural Sciences, Kangwon National University, 24341 Chuncheon, South Korea

**Keywords:** Acute lung injury, STAT1, IRF1, Fludarabine, Autophagy, Inflammation

## Abstract

**Background:**

Acute lung injury (ALI), including its most severe form, acute respiratory distress syndrome (ARDS), is a common cause of acute hypoxemic respiratory failure. Although its clinical characteristics have been well characterized, the relevant mechanism remains unclear. An imbalance in autophagy leads to alveolar remodeling and triggers the pathogenesis of ARDS. In this study, we assessed the therapeutic efficacy of the STAT1 inhibitor fludarabine (Fluda) in ALI. C57BL6 mice were exposed to lipopolysaccharide (LPS), and their lung tissues were analyzed via next-generation transcriptome sequencing.

**Results:**

Western blotting revealed that interferon regulatory factor 1 (IRF1) was highly expressed and STAT1 was phosphorylated following LPS exposure. Fluda significantly decreased the protein expression of STAT1/IRF1 and inhibited the alveolar infiltration of neutrophils and macrophages. Nitric oxide (NO), inducible nitric oxide synthase, tumor necrosis factor-α (TNF-α), interferon-γ, and interleukin-6 (IL-6) release was decreased in the lungs of mice and RAW264.7 macrophages following Fluda treatment. In LPS-induced GFP-LC3 transgenic mice treated with Fluda, the counts of LC3-expressing neutrophils and macrophages in bronchoalveolar (BAL) fluid were significantly decreased. Furthermore, Fluda decreased LC3 and p62 protein expression, thereby inhibiting the release of NO, IL-6, and TNF-α in BAL. In RAW264.7 cells, the inhibition of STAT1/IRF1 by Fluda decreased LPS-induced ERK and NF-κB p65 phosphorylation.

**Conclusions:**

The inhibition of STAT1/IRF1 by Fluda plays a pivotal role in modulating dysregulated autophagy by suppressing the MAPK and NF-κB p65 pathways in ALI.

**Supplementary Information:**

The online version contains supplementary material available at 10.1186/s42826-025-00245-7.

## Introduction

Acute lung injury (ALI) is characterized by rapid-onset microvascular protein leakage into the alveolar space, inflammatory cell infiltration, interstitial edema, and disruption of epithelial integrity, followed by severe lung damage [1]. ALI is clinically defined as acute respiratory distress syndrome (ARDS), a devastating condition with high mortality rates [2]. Despite significant advances, drugs for ARDS treatment are lacking [3]. Several emerging risk factors for ALI have been proposed; however, its pathophysiology involves a complicated imbalance among proinflammatory and anti-inflammatory cytokines, oxidants, and antioxidants. Lipopolysaccharide (LPS), a well-known endotoxin, elicits immune responses by promoting the secretion of proinflammatory cytokines and inflammatory mediators, such as tumor necrosis factor-α (TNF-α), interleukin-6 (IL-6), and nitric oxide (NO) [1]. In the context of innate immune responses in ALI, alveolar macrophages, neutrophils, and dendritic cells play important roles in initiating multiple immunologic processes and tissue injury in ALI [4, 5]. It was recently demonstrated that endotoxin induces abnormal autophagic responses in innate immune cells [6–8]. Autophagy is a tightly regulated process that facilitates the degradation of overabundant or malfunctioning cellular components through the formation of autophagosomes [9, 10]. Several molecular mechanisms are involved in autophagosome biogenesis. During autophagy, LC3B-I is converted into LC3B-II, leading to the elongation of autophagosomal membranes, and the interactions of p62 and SQSTM1 with LC3B facilitate the degradation of ubiquitinated proteins [11–13]. Although the abundance of autophagosomes with increased autophagic activity may mislead to autophagic processes, it is a largely unknown LC3/p62-mediated cellular pathway in ALI.

The JAK/STAT pathway has been found to play vital roles in initiating immune responses [14]. STAT1 can be activated by its upstream phosphorylated molecules JAK1 and JAK2. Activated STAT1 is transported into the nucleus, where it promotes the production of proinflammatory factors, including TNF-α and IL-1 [15, 16]. STAT1 activation is critically involved in the early steps of LPS-induced pulmonary inflammation, gastric acid aspiration, and acute pancreatitis-induced ALI [17].

The interferon regulatory factor (IRF) family consists of nine members (IRF1–9) that contain a helix–turn–helix N-terminal DNA-binding domain and a regulatory C-terminal portion that mediates interactions between different IRFs and between IRFs and other transcription factors [18]. IRFs play critical roles in the regulation of immune transcription factors involved in the regulation of immune processes and oncogenesis [19–24]. It has been reported that inflammation was significantly reduced during sepsis in IRF1-knockout mice [25, 26]. However, the mechanism by which IRF1 regulates autophagy in ALI remains unknown.

In this study, we demonstrated that IRF1 is an early proinflammatory signal in LPS-induced ALI. Furthermore, we determined that the inhibition of STAT1 by fludarabine (Fluda) and its downstream transcriptional target IRF1 suppressed LPS-induced inflammatory responses and dysregulated autophagic responses in macrophages in vitro and in vivo. Therefore, these findings suggest that STAT1/IRF1 inhibition is a feasible strategy for ALI treatment.

## Methods

### Mice

Ten-week-old male C57BL/6 mice were purchased from Doo Yeol Biotech (Seoul, Korea). To analyze autophagic puncta, we used GFP^*tg*^-LC3 mice (#53, RBRC00806) obtained from RIKEN (Wako, Japan). These mice are congenic with the C57BL/6 J line. Studies involving the usage of mice were approved by the Institutional Animal Care and Use Committee (IACUC, KW-210203–2, Kangwon National University, Korea) and conducted in accordance with the IACUC regulations.

### Murine model of LPS-induced ALI

Mice were divided into the control, Fluda, LPS, and LPS with Fluda groups (n = 6/group). Mice were anesthetized with Zoletil 50 (Virbac, Carros, France; 30 mg/kg, i.p.) and challenged via the intratracheal administration of 50 µL saline or 1 mg/kg LPS (*Escherichia coli*, O111:B4; Sigma-Aldrich, St. Louis, MO, USA) dissolved in 50 µL of saline [27]. In the LPS with Fluda group, mice were challenged via the intratracheal administration of 15 mg/kg Fluda (Selleck Chemicals, Houston, TX, USA) 4 h before LPS administration. Mice in the control and Fluda groups were injected with saline and Fluda, respectively, to serve as negative and positive controls for inflammatory responses induced by Fluda. At 24 h after LPS administration, mice were sacrificed. Fluda and/or LPS administration did not affect mouse survival rates.

### RNA preparation and transcriptome sequencing

Total RNA was isolated from the lungs of control mice and mice with LPS-induced ALI using a GeneAll^R^ RiboEx Total RNA extraction kit (GeneAll Biotechnology, Seoul, Korea). Libraries were constructed and sequenced at Macrogen Inc. (Seoul, Korea) using their standard procedure.

### Quality control and data management

For quality control, read quality was verified by FastQC, and read alignment was verified by Picard. Differential gene expression analysis was performed using TopHat and Cufflinks software [27]. The quantities of isoforms and gene transcripts were computed, and transcript abundance was assessed in fragments per kilobase of exon per million fragments mapped (FPKM). Expression was derived as FPKM values for each gene in each sample using Cufflinks software. Genes with FPKM values of 0 across all samples were excluded. The filtered dataset was subjected to upper quantile normalization. Statistical significance was determined using Student’s *t*-test, and the false discovery rate was controlled by adjusting P values using the Benjamini–Hochberg method [28].

### Reverse transcription-polymerase chain reaction (PCR)

Total RNA was isolated from cells and lung tissue using a GeneAll® RiboEx Total RNA extraction kit (GeneAll Biotechnology, Seoul, Korea). cDNA was synthesized using Reverse Transcription Master Premix (Elpis Biotech, Daejeon, Korea; EBT-1516) [29]. The cDNA was then amplified in a reaction mixture containing 10 pmol of primers and 1 mg of total cDNA using AccuPower PCR PreMix (Bioneer, Seoul, Korea; K-2080–3). The primer pairs are presented in Table 1, and the amplification conditions were as follows: predenaturation at 94 °C for 5 min; 30 cycles of denaturation at 94 °C for 30 s, annealing at 53 °C–60 °C for 30 s, and elongation at 72 °C for 1 min [30]; and a final elongation step at 72 °C for 10 min.

**Table 1:** Sequences of the primers in real-time PCR analysis

Species	Genes	Sequence
Mouse	*Nos2*	F:5'-GGATCTTCCCAGGCAACCA-3'
		R:5'-TCCACAACTCGCTCCAAGATT-3'
	*Beclin-1*	F:5'-ATGGAGGGGTCTAAGGCGTC-3'
		R: 5'-TGGGCTGTGGTAAGTAATGGA-3'
	*Gapdh*	F:5'- GGCAAATTCAACGGCACAGT −3'
		R:5'- CGCTCCTGGAAGATGGTGAT −3'

### Harvesting of lungs and bronchoalveolar lavage fluid (BALF)

Twenty-four hours after LPS administration, the left lung of each mouse was snap-frozen and subsequently processed to obtain lung homogenate. To isolate BALF, mice were sacrificed, and their tracheas were immediately lavaged twice with 1 mL of ice-cold PBS via a catheter. Bronchoalveolar lavage (BAL) cells were collected via centrifugation at 2000 rpm for 5 min at 4 °C. The collected supernatant was used for enzyme-linked immunosorbent assay (ELISA). BAL cells (1 × 10^6^ cells/mL in ice-cold PBS) were fixed with methanol and stained with Hema-3 stain (Thermo Fisher Scientific, Waltham, MA, USA) according to the manufacturer’s instructions. Cells were counted under a light microscope.

### Fluda treatment and LPS stimulation of RAW264.7 cells

RAW264.7 cells, derived from murine macrophages, were obtained from the Korea Cell Line Bank (Seoul, Korea) and grown in RPMI 1640 medium containing 10% fetal bovine serum in a 37 °C humidified incubator. Cells were pretreated with 20 μM Fluda for 1 h, followed by cotreatment with 100 ng/mL LPS and 20 μM Fluda. We collected cells or their supernatant for subsequent analysis after 24 h of LPS treatment.

### Measurement of myeloperoxidase (MPO) activity

MPO activity was measured using a previously described method. In brief, lung tissue was homogenized in 0.5% hexadecyltrimethylammonium bromide in 50 mM potassium phosphate buffer (pH 6.0) [31]. The homogenate was centrifuged at 12,000 rpm for 10 min at 4 °C. In the supernatant, the H_2_O_2_-dependent oxidation of *o*-dianisidine solution (3,3′-dimethoxybenzidine dihydrochloride in potassium phosphate buffer, pH 6.0) was measured at 450 nm using an Epoch microplate spectrophotometer (BioTek, Winooski, VT, USA).

#### ELISA

Inflammatory cytokine levels were measured in conditioned medium or BALF using Duoset ELISA development kits (R&D Systems, Minneapolis, MN, USA) according to the manufacturer’s instructions.

### Histopathological analysis

For histological analysis, the left lung lobes were trimmed and fixed in formalin for 24 h. Paraffin-embedded tissues were cut into 4-μm sections and stained with hematoxylin and eosin. The lung injury score was calculated as the sum of the scores for alveolar wall thickening walls, neutrophil infiltration, and intra-alveolar hemorrhage [32]. Each histological characteristic was scored from 0 to 4.

### Western blotting

Whole-cell lysates were prepared in RIPA lysis buffer. In total, 20 μg of proteins were mixed with 5 × SDS-PAGE sample buffer (Tech & Innovation, Chuncheon, Korea; BSS-9005), electrophoresed through a 10% polyacrylamide gel, and transferred to a 0.45-mm nitrocellulose transfer membrane (Bio-Rad, Hercules, CA, USA; 162–0115) [32]. For blocking, membranes were incubated with 5% skim milk and incubated with primary antibodies for overnight at 4 °C. Primary antibodies against pSTAT1 (Ser727), pSTAT1 (Tyr701), STAT1, IRF1, IRF7, LC3B, and β-actin were purchased from Cell Signaling Technology, (Danvers, MA, USA). Primary antibodies against p62 and inducible nitric oxide synthase (iNOS) were purchased from Santa Cruz Biotechnology (Dallas, TX, USA). After washing, the membranes were incubated with HRP-conjugated secondary antibodies for 1 h at room temperature. Subsequently, the protein bands were detected using ECL detection solution.

### NO production

NO production was detected using the modified Griess method [33]. NaNO_2_ solution was used to create a standard curve. Conditioned medium (cell culture supernatant), lung BALF, and standard samples were mixed with equal volumes of 1 × Griess reagent (G4410; Sigma-Aldrich) at room temperature. Next, the absorbance at 540 nm was measured using a microplate reader (BioTek Inc., WA, USA).

### *Immunofluorescence staining for GFP*.^*tg*^*-LC3*

In GFP^*tg*^-LC3 mice, BALF was collected and centrifuged to harvest cells. The cells were spun down in a slide using a Cytospin centrifuge. GFP^*tg*^-LC3 was detected using anti-GFP antibody (Santa Cruz Biotechnology). After incubation with primary antibodies, Alexa Fluor 488-labeled anti-rabbit secondary antibodies were used. Then, Fluoroshield Mounting Medium with DAPI solution was used to label the nuclei. As a negative control, we harvested BALF cells from C57BL/6 wild-type mice and stained them in a similar manner without primary antibody. All samples were visualized under an Olympus FluoView FV1000 confocal laser-scanning microscope (Olympus, Tokyo, Japan) at 488 nm.

### Statistical analysis

All statistical data were analyzed using GraphPad Prism 9.0 (GraphPad Software, Boston, MA, USA). The results are expressed as mean ± SD. Comparisons among multiple groups were performed using Bonferroni’s multiple comparison test. A *p* value of < 0.05 was considered statistically significant. The experiment was conducted three times with three replicates.

## Results

### IRF1 expression was increased during the early phase of LPS-induced ALI

**Fig. 1:**
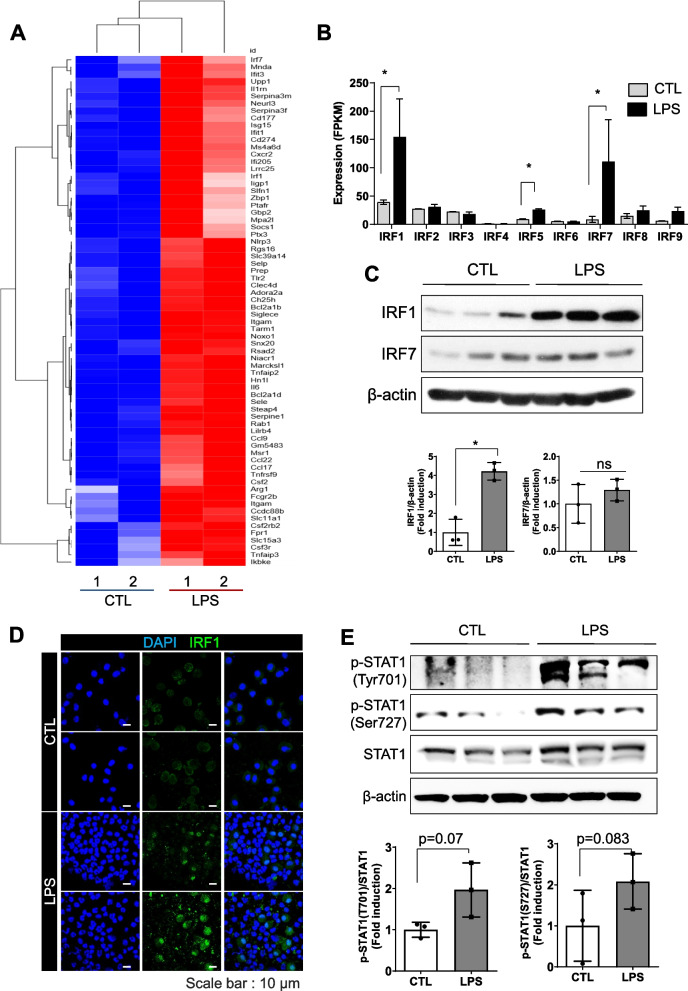
The STAT1/IRF1 pathway is associated with LPS-induced ALI in mice. **A** RNA-seq data presenting comparisons of the expression of several genes, including IRF1 family genes, between the control and LPS groups of mouse lung tissue lysates (*n* = 2 per group). **B** Expression of IRF family genes was measured as FPKM values. **C** Western blotting of IRF1 and IRF7 in mouse lung tissue (upper) and their densitometric ratios against β-actin (bottom) (*n* = 3 per group). *p < 0.05 compared with the control group. ns: not significant. **D** Confocal images of immunostaining for IRF1 (green) in BAL cells. Scale bar, 10 μm (*n* = 2 per group). **E** Western blotting of pSTAT1 (Tyr701), pSTAT1 (Ser727), total STAT1, and β-actin in mouse lung tissue (upper) and their densitometric ratios against STAT1 (bottom) (*n* = 3 per group). The data are presented as mean ± SD

To determine the relationships of inflammatory processes with ALI at the molecular level, we sequenced the transcriptome in lungs of mice with LPS-induced ALI using RNA sequencing (RNA-seq). The heatmap of RNA-seq expression data revealed that 70 genes were upregulated following LPS exposure in mice lungs (Fig. 1A, Fig. S1). In particular, the expression of various members of the IRF family was considerably altered. IRFs play critical roles in immune responses and host defenses [18]. Therefore, to further explore differences in the expression of IRFs between the LPS-induced ALI and control groups, we calculated their average FPKM values (Fig. 1B). The expression of IRF1 and IRF7 was significantly higher in mice with LPS-induced ALI than in control mice (saline-treated mice). We next determined the protein expression of IRF1 and IRF7 to validate the RNA-seq data. IRF1 expression, but not IRF7 expression, was significantly increased in LPS-treated mice compared with that in untreated mice (Fig. 1C). Moreover, we investigated IRF1 expression in BAL cells. As presented in Fig. 1D, IRF1 was highly expressed in BAL cells, especially in the nuclei of macrophage-like cells, which have one large nucleus, distinguishing them from neutrophils (Fig. 1D). Because IRF1 is a well-known downstream target of interferon (IFN)/STAT1 [34], we next determined whether LPS-induced IRF1 was associated with STAT1 signaling. Western blotting revealed that the expression of STAT1 phosphorylated at both Tyr701 and Ser727 was highly increased in the lung tissue of LPS-treated mice compared with that in control mice (Fig. 1E).

### Fluda reduced STAT1 activation and IRF1 expression in LPS-injected mice

**Fig. 2:**
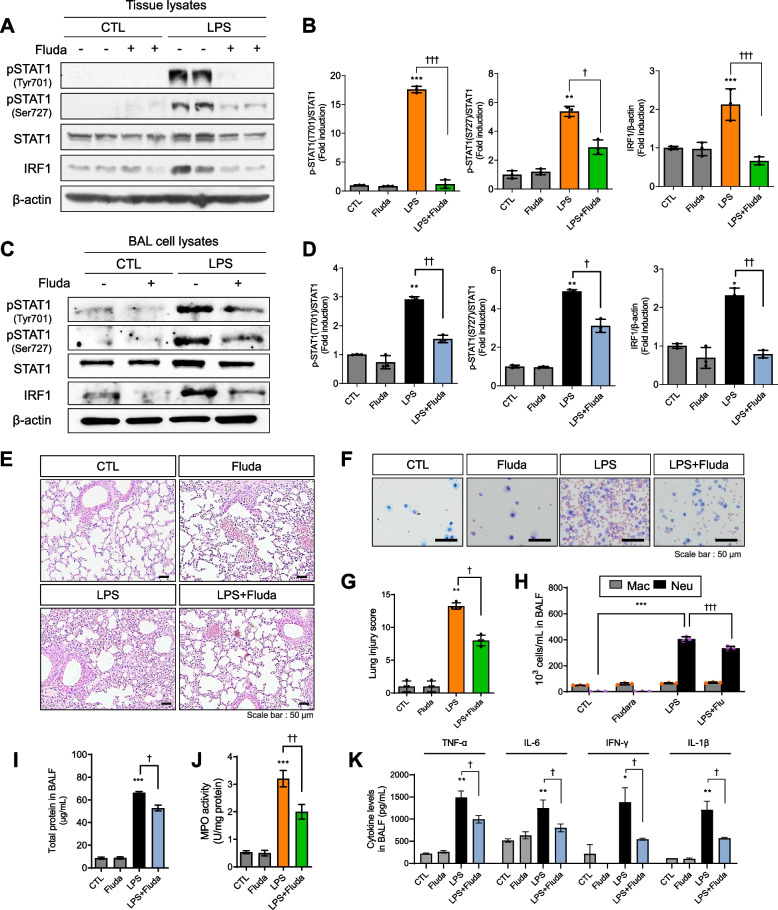
The STAT1/IRF1 inhibitor Fluda attenuates LPS-induced ALI in mice. **A** pSTAT1 (Tyr701), pSTAT1 (Ser727), STAT1, IRF1, and β-actin were detected in mouse lung tissue. **B** Densitometric ratios of pSTAT1 (Tyr701) and pSTAT1 (Ser727) against STAT1 and that of IRF1 against β-actin. **C** pSTAT1 (Tyr701), pSTAT1 (Ser727), STAT1, IRF1, and β-actin expression was detected in BAL cell lysates using western blotting. **D** Densitometric ratios of pSTAT1 (Tyr701) and pSTAT1 (Ser727) against STAT1 and that of IRF1 against β-actin (*n* = 3). **E** Representative images of hematoxylin and eosin-stained lung sections from four experimental groups (× 200). Scale bar, 50 μm. **F** BAL cells were subjected to Giemsa staining and then observed under a microscope (× 200). Scale bar, 50 μm. **G** The lung injury score illustrated Fluda reduced LPS-induced ALI in mice (*n* = 4 per group). **H** The numbers of macrophages and neutrophils in BALF. **I** Total protein in BALF was measured using the BCA assay. **J** MPO activity was measured in whole-lung lysates. **K** Inflammatory cytokines in BALF, including TNF-α, IL-6, IFN-γ, and IL-1β, were detected using ELISA. The data are presented as mean ± SD, *n* = 3 independent experiments were performed. *p < 0.05, **p < 0.01, and ***p < 0.001 compared with the control group. †*p* < 0.05, ††*p* < 0.01, and †††*p* < 0.001 compared with the LPS group

Because STAT1/IRF1 signaling was activated following LPS exposure, we intratracheally injected Fluda, an inhibitor of STAT1, into LPS-exposed mice to investigate the pathological changes induced by LPS under the abrogation of STAT1/IRF1 signaling [35]. First, we investigated whether Fluda inhibited IRF1/STAT1 signaling in LPS-exposed mice. STAT1 and IRF1 phosphorylation was successfully inhibited by Fluda in lung tissue homogenates (Fig. 2A, 2B). Based on our finding that IRF1 was remarkably expressed on inflammatory cells in BALF (Fig. 1D, 1E), we explored IRF1/STAT1 expression in BAL cells. Consistent with the immunofluorescence data, LPS increased IRF1/STAT1 levels in BAL cell lysates, whereas Fluda effectively inhibited IRF1/STAT1 (Fig. 2C, 2D).

### Fluda attenuated LPS-induced tissue injury and inflammation in mice

Lung tissues exposed to LPS were significantly damaged, and a large number of inflammatory cells, alveolar septic thickening, and interstitial edema were observed. However, these changes were obviously relieved by pretreatment with Fluda (Fig. 2E, 2G). Indeed, Fluda inhibited the LPS-mediated infiltration of inflammatory cells, including neutrophils and macrophages, into the lungs (Fig. 2F, 2H). Moreover, LPS exposure markedly increased the protein concentration, suggesting an increase in alveolar permeability, whereas Fluda prevented lung permeability following LPS exposure (Fig. 2I). In addition, alveolar MPO activity was significantly elevated in the LPS group; however, Fluda significantly decreased MPO activity (Fig. 2J). In BALF, LPS increased the levels of proinflammatory cytokines, including TNF-α, IL-6, IFN-γ, and IL-1β, whereas Fluda reduced these levels (Fig. 2K). Overall, these data suggested that Fluda could alleviate LPS-induced ALI by suppressing STAT1/IRF1 signaling.

### Fluda suppressed iNOS expression and phosphrylated NF-κB/ERK1/2 signaling in mice

**Fig. 3:**
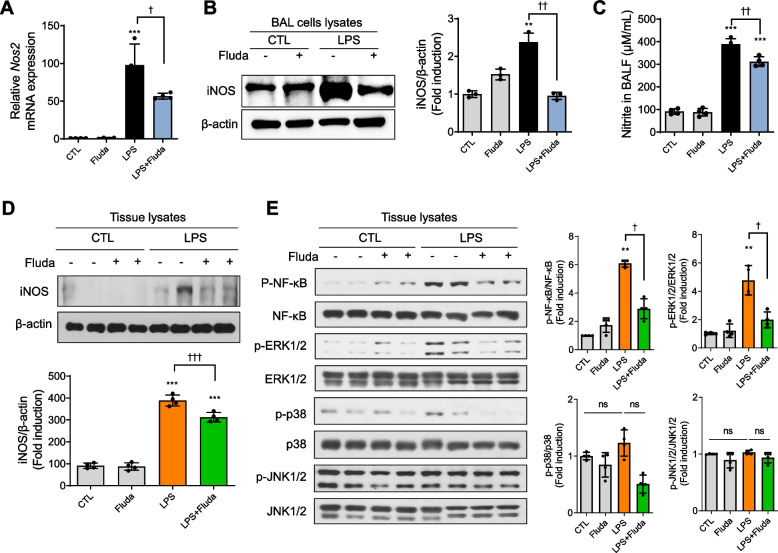
Fluda inhibited iNOS, NF-κB, and ERK signaling. **A** The mRNA expression of *Nos2* was detected in BAL cell lysates using real-time PCR. **B** The protein expression of iNOS was detected using western blotting (left). Densitometric ratio of iNOS against β-actin (right). **C** Release of NO was determined by measuring the amount of nitrite in BALF using Griess reagent. **D** iNOS protein expression in lung tissue was detected using western blotting (upper). Densitometry ratio of iNOS in tissue lysates against β-actin (bottom). **E** The expression of phosphorylated NF-κB (Ser536), JNK1/2, ERK1/2, and p38 was detected. Total NF-κB, total JNK1/2, total ERK1/2, and total p38 were used as loading controls. The proteins were isolated from mice lung tissue. Densitometric ratios of pNF-κB, pERK1/2, pJNK1/2, and p-p38 in tissue lysates against loading controls. The data are presented as mean ± SD (n = 3 per group for A-C and n = 4 per group for D and E). **p < 0.01, and ***p < 0.001 compared with the control group. †*p* < 0.05, ††*p* < 0.01, and †††*p* < 0.001 compared with the LPS group. ns: not significant

As NO is a major inflammatory mediator involved in endotoxin-induced inflammatory responses, we determined whether Fluda modulated iNOS expression and NO production in LPS-injected mice. In acute inflammatory responses, both iNOS and NO are produced by various immune cells, such as macrophages and neutrophils [36]. Fluda treatment significantly inhibited iNOS at both the mRNA and protein levels in BAL cells (Fig. 3A, 3B). In addition, we measured nitrite levels in BALF using the Griess assay, which revealed that NO production was reduced by Fluda treatment (Fig. 3C). We further measured iNOS expression in whole-lung tissue and found that it was slightly but not significantly reduced by Fluda treatment (Fig. 3D). These data demonstrated that Fluda reduces iNOS signaling in macrophages in the lungs.

Furthermore, the inhibition of STAT1/IRF1 signaling by Fluda led to diminished pNF-κB p65 and pERK1/2 expression compared with the findings in mice treated with LPS alone (Fig. 3E). p-p38 expression tended to be reduced by Fluda, albeit without significance, whereas pJNK1/2 expression was not altered by LPS or Fluda exposure. These findings illustrated that Fluda inhibits LPS-induced increases in iNOS and p-NF-κB/p-ERK1/2 expression in mice with ALI.

### Fluda inhibited dysregulated autophagy in LPS-exposed inflammatory cells in the mouse lung

**Fig. 4:**
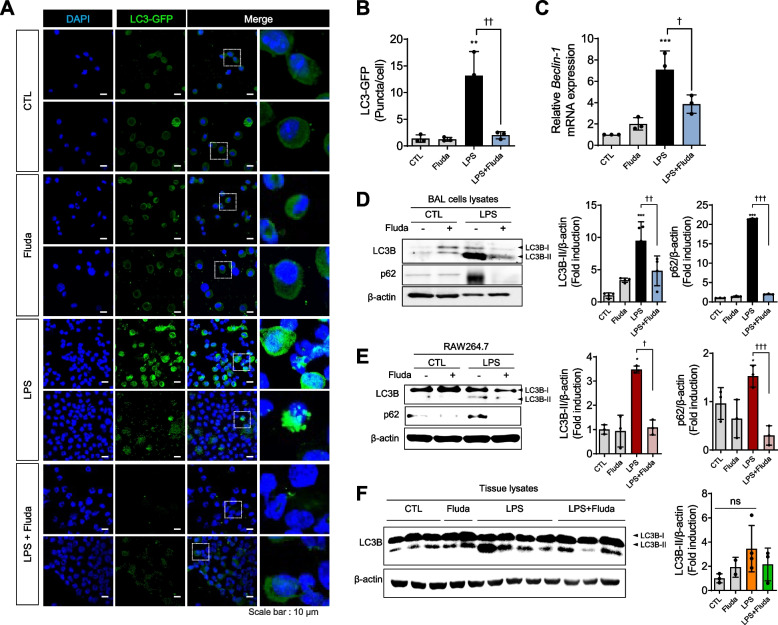
Fluda reduced LPS-mediated abnormal autophagic responses in BAL cells at 24 h after LPS administration in GFP-LC3 transgenic mice. **A** Representative images of immunofluorescence staining for GFP-tagged LC3B. Confocal microscopy (× 1200) was used to monitor LC3-dependent autophagy. Scale bar, 10 μm. **B** The average number of LC3-GFP puncta per cell was counted. **C** In BAL cell lysate, the mRNA expression of beclin-1 was determined using real-time PCR. **D** LC3B, p62, and β-actin expression in BAL cells was determined using western blotting (left). Densitometric ratio of LC3B-II in BAL cells against β-actin (middle) and that of p62 in BAL cell lysates against β-actin (right). **E** The protein expression of LC3B and p62 was detected in RAW264.7 cells. Densitometric ratio of LC3B-II in RAW264.7 against β-actin (middle) and that of p62 in RAW264.7 β-actin (right). **F** LC3B and β-actin expression in whole-lung lysates was determined using western blotting (left). Densitometric ratio of LC3B-II in whole-lung lysates against β-actin (right) (*n* = 3 for CTL, n = 2 for Fluda, n = 4 for LPS, and *n* = 3 for LPS + Fluda). The data are presented as mean ± SD (*n* = 3 per group). *p < 0.05, **p < 0.01, and ***p < 0.001 compared with the control group. †*p* < 0.05, ††*p* < 0.01, and †††*p* < 0.001 compared with the LPS group

Because autophagy is strongly related to various inflammatory diseases, we examined the role of autophagy in LPS-induced ALI and the effect of Fluda on autophagic activity. Autophagic activity in BAL cells in GFP-LC3 transgenic mice was assessed to visualize and quantify functional autophagy by detecting GFP levels in living BAL cells. Cytoplasmic puncta, which contained autophagosomes, were strongly increased by LPS exposure, whereas Fluda pretreatment reduced their appearance in LPS-treated mice (Fig. 4A, 4B). Moreover, the expression of Beclin-1, an essential mediator of autophagy [37], was regulated by Fluda under LPS-induced injury, suggesting that Fluda could abolish autophagy (Fig. 4C). We measured the conversion of LC3B-I to LC3B-II, an indicator of autophagy, in lung tissue, BAL cells, and murine macrophages (RAW264.7) via immunoblotting. LC3B-II expression was significantly increased after LPS exposure, and Fluda decreased LC3B-II protein expression in BAL cells (Fig. 4D). In addition, Fluda treatment effectively reversed the excessive accumulation of p62 (Fig. 4D). We next determined whether the autophagic response in macrophages in vivo was consistent with that in BAL cells in mice. In RAW264.7 cells, LC3B-II and p62 expression was increased by LPS but inhibited by Fluda (Fig. 4E). Conversely, in lung tissue homogenates, LC3B-II expression was slightly changed following treatment, suggesting that autophagy mainly occurs in inflammatory cells (Fig. 4F). These data indicated that LPS induced dysregulated autophagy, leading to p62 accumulation, whereas Fluda treatment modulated abnormal and excessive autophagic responses mainly in inflammatory cells.

### Fluda diminished phosphorylated STAT1/IRF1, iNOS, and phosphorylated NF-κB/ERK1/2 signaling in macrophages

**Fig. 5:**
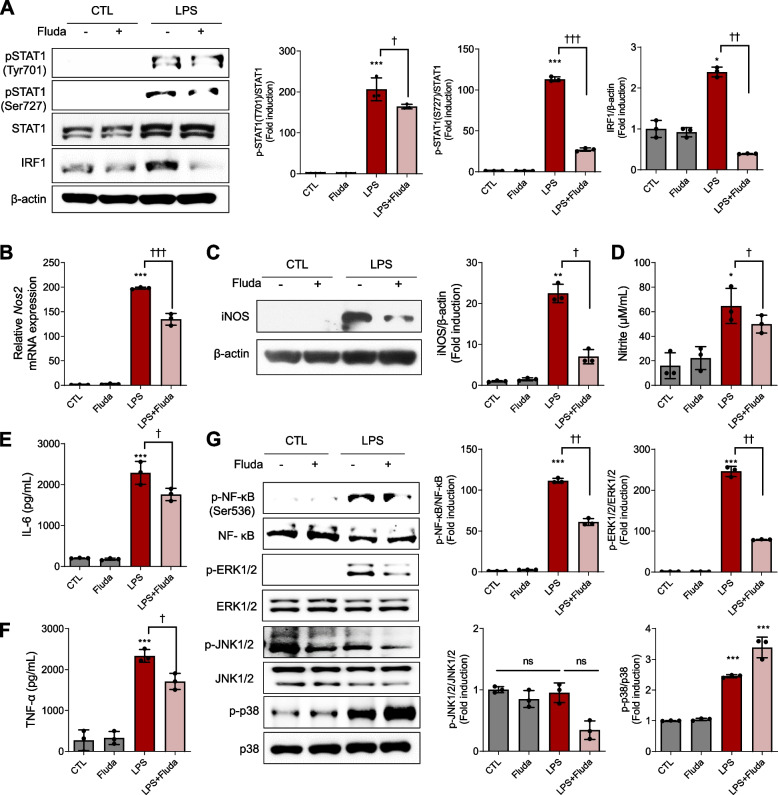
STAT1/IRF1, iNOS, and NF-κB/ERK1/2 activation was attenuated by Fluda in RAW264.7 cells. **A** pSTAT1 (Tyr701), pSTAT1 (Ser727), IRF1, and STAT1 were detected in RAW264.7 cells. STAT1 and β-actin were used as loading controls. Densitometric ratios of pSTAT1 (Tyr701) and pSTAT1 (Ser727) against STAT1 and that of IRF1 against β-actin. **B** Relative *Nos2* expression in RAW264.7 cells was measured using real-time PCR. **C** Western blotting for iNOS expression. Densitometric ratio of iNOS against β-actin. **D** NO release was determined by measuring the amount of nitrite in conditioned medium using Griess reagent. **E** IL-6 and (**F**) TNF-α levels in conditioned medium were detected using ELISA. **G** Western blotting for p-NF-κB (Ser536), p-ERK1/2, p-JNK1/2, and p-p38. Total NF-κB, total JNK1/2, total ERK1/2, total p38, and β-actin were used as loading controls. All blots were subjected to densitometric analysis and relative quantification. Data are presented as mean ± SD (*n* = 3 per group). *p < 0.05, **p < 0.01, and ***p < 0.001 compared with the control group. †*p* < 0.05, ††*p* < 0.01, and †††*p* < 0.001 compared with the LPS group. ns: not significant

As we observed p-STAT1/IRF1 and p-NF-κB/p-ERK1/2 upregulations following LPS injection in mice, we validated the cellular pathways in vitro. Fluda efficiently inhibited p-STAT1/IRF1 signaling in RAW264.7 cells (Fig. 5A). Furthermore, Fluda reduced LPS-mediated iNOS signaling (Fig. 5B‒5D) and the release of inflammatory cytokines, including IL-6 and TNF-α, in RAW264.7 cells (Fig. 5E, 5 F). Fluda suppressed p-NF-κB and p-ERK1/2 activation (Fig. 5G) but not p-JNK1/2 and p-p38, consistent with our in vivo data (Figs. 3E, 5G). These findings illustrated that Fluda effectively inhibited LPS-mediated p-STAT1/IRF1 signaling with decreased p-NF-κB/p-ERK1/2 activation and regulated autophagy-related inflammation.

## Discussion

In this study, we demonstrated that the p-STAT1/IRF1 pathway, which is associated with intrinsic autophagy, is critical for ALI. RNA-seq revealed significant differential expression of IRFs in LPS-exposed mouse lungs. IRFs play essential roles in the development and functioning of innate immune cells [38]. In particular, IRF1 expression was remarkably increased in lung tissues including neutrophils and macrophages. Wo and colleagues reported that the absence of IRF1 abrogates pyroptosis in alveolar macrophages and alleviates alveolar injury and inflammation in mice. In addition, they demonstrated the importance of IRF1 in controlling caspase-1–dependent inflammation in macrophages [39]. Alveolar macrophages comprise 90% of the cells in BALF, and they serve as an air–blood barrier against a multitude of microorganisms and airborne particles. Thus, the regulation of macrophages plays a pivotal role in the pathogenesis of ALI/ARDS. Autophagy is a self-degradative process characterized by the formation of autophagosomes and their fusion with lysosomes [40, 41]. In macrophages, autophagy participates in cellular homeostasis by enabling the removal of intracellular microorganisms and pathogens for degradation. Recently, the controversial role of autophagy in various inflammatory diseases has emerged [8, 42, 43]. It has been suggested that autophagy positively regulates inflammatory responses in ARDS/ALI [44, 45], whereas acute injury induces an dysregulated autophagic response, leading to negative exacerbation [46]. Although autophagy is considered a lysosomal degradative process in antibacterial defenses, dysregulated autophagy is associated with abnormal cell death or damage [47]. To assess autophagy in cells, immunoblotting and immunohistochemical staining are widely used to detect LC3 [48–50]. During autophagy, LC3B-I, a cytosolic form, is conjugated to phosphatidylethanolamine to form LC3B-II, which is associated with the internal and external autophagosome membranes [51]. In this process, p62, an adaptor protein, serves as a selective substrate of autophagy. Specifically, it delivers ubiquitinated proteins or bacteria to autophagosomes, binds LC3, and is degraded in the autolysosome [52]. Hence, it has been reported that enhanced levels of LC3B-II and decreased levels of p62 are the primary indicators of normal autophagic flux [53]. Using GFP-LC3^tg^ mice in this study, we observed that LPS increased LC3 puncta in BAL cells, especially macrophages. Moreover, immunoblotting data illustrated that LPS induced the accumulation of p62 and LC3B-II in BAL cells. Yoshii and colleague reported that bafilomycin A1, a potent V-ATPase inhibitor, induces the accumulation of LC3-II and p62 by inhibiting lysosomal degradation, and the expression of both proteins was increased on immunoblotting [48]. Consistent with their findings, we observed that LPS increased LC3B-II and p62 expression and Fluda facilitated their degradation under LPS stimulation in our mouse model. Therefore, these findings suggested that LPS exposure mediated the excessive formation of autophagosomes in lung immune cells, but it did not increase degradation within autolysosomes. In other words, our findings suggested that LPS exposure interfered with lysosomal degradation during dysregulated autophagy, leading to the accumulation of both LC3B-II and p62 without autophagic degradation. In this context, it is notable that the inhibition of the STAT1/IRF1 pathway led to significant p62, LC3B-II, and beclin-1 degradation in BAL cells. Beclin-1 is a central regulator of autophagy, and it forms a multimeric complex with vacuolar protein sorting 34 and class 3 phosphatidylinositol 3-kinase. This complex is necessary for autophagosome biogenesis [54]. Bacterial clearance by macrophages via phagocytosis is the first line of the host defense system to eliminate invading pathogens. Fernandez and colleagues validated that bacterial activity is enhanced through the disruption of beclin-1/Bcl-2 binding, leading to increased basal autophagy in Becn1^F121 A^ mice [55]. In the alveolar macrophages of *atg7* KO mice, Atg7 deficiency altered the phagocytic activity of *Klebsiella pneumoniae* and markedly increased the levels of proinflammatory cytokines. Consistent with these studies, we observed that LC3B-II and p62 expression was increased in response to LPS, and treatment with Fluda significantly reduced their expression. Furthermore, the excessive activation of autophagy in alveolar macrophages and inhibition of the STAT1/IRF1 axis regulate autophagic activation in mice with ALI.

STAT1 has been implicated as a mediator of biological responses, and it is transiently activated in response to ligand stimulation. STAT1 activation modifies the transcription of a fraction of IFN-γ–sensitive genes. IFN-γ is the representative macrophage-activating cytokine in inflammatory processes that functions via innate lymphocytes or T lymphocytes. The binding of IFN-γ to IRF1 receptor activates JAK1/JAK2, leading to phosphorylation and interactions between STAT1 homodimers and GAS elements. In addition, the STAT1/IRF1 axis plays a major role in controlling macrophage transcription upon IFN-γ activation. We found that the inhibition of the STAT1/IRF1 axis by Fluda reduced host inflammatory and antimicrobial defenses in an ALI experimental model. Fluda is a fluorinated purine nucleoside analog that is used in various hematological malignancies [56, 57], and it is a STAT1 inhibitor. In this study, Fludarabine significantly reduced neutrophil counts and MPO activity in LPS-induced acute lung injury, suggesting its role in attenuating neutrophil activation and inflammation. However, the reduction was not dramatic, which may suggest that its effects could vary depending on timing or dose. These findings provide insight into fludarabine’s potential as an anti-inflammatory agent, warranting further investigation. Moreover, the inhibition of STAT1/IRF1 signaling by Fluda decreased LPS-induced increases in iNOS, NF-κB, and ERK1/2 signaling as well as NO production in BALF. In general, NO accumulation is observed in the lungs of patients with ARDS, and it is associated with iNOS expression in alveolar macrophages [58]. Furthermore, the activation of NF-κB and ERK1/2 signaling is related to inflammation in patients with ARDS [59, 60]. All these previous reports indicate that Fluda has inhibitory effects on iNOS, NF-κB, and ERK1/2 signaling, thereby having a considerable clinical value for patients with ALI/ARDS. It has been reported that IRF1 expression is increased in mice with LPS-induced ARDS/ALI, and efforts to attenuate ALI by neutralizing IRF1 in knockout mouse models have been attempted for therapeutic approaches. Nevertheless, in this study, we investigated the therapeutic effect of Fluda through the inhibition of STAT1/IRF1 signaling upon the intratracheal instillation of endotoxin to mice.

## Conclusions

**Fig. 6:**
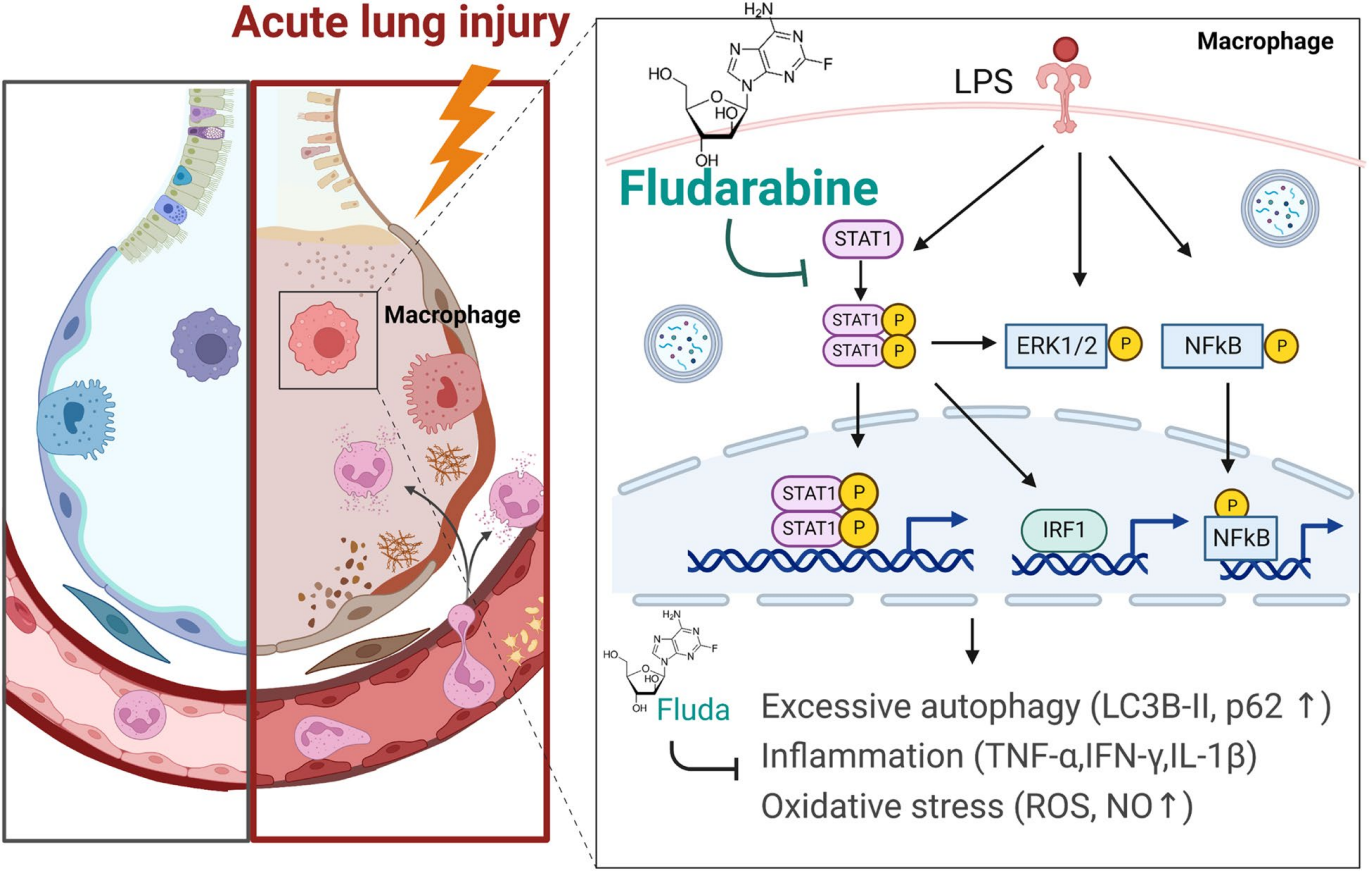
The protective effect of Fluda in mice with LPS-induced ALI. LPS increased iNOS expression and NO release and enhanced STAT1/IRF1, ERK1/2, and NF-κB signaling, which were relevant to the dysregulated autophagy in alveolar macrophages. Fluda effectively decreased downstream signaling induced by LPS under inflammatory conditions

In this study, we determined that STAT1/IRF1 inhibition by Fluda decreased the number of infiltrating inflammatory cells, reduced inflammatory cytokine levels, and suppressed iNOS expression by inhibiting the NF-κB/ERK1/2 pathways. We further demonstrated that treatment with Fluda reduced LPS-induced impairments in autophagic flux in alveolar macrophages, which could explain the activation of the STAT1/IRF1 axis. Therefore, these findings suggest that Fluda could have therapeutic utility for ALI and autophagy-associated human diseases (Fig. 6).

## Supplementary Information

Additional file 1.

## Data Availability

The datasets used and/or analyzed during the current study are available from the corresponding author on reasonable request.

## Abbreviations

ALI: Acute lung injury
ARDS: Acute respiratory distress syndrome
Fluda: Fludarabine
STAT: Signal transducer and activator of transcription
IRF: Interferon regulatory factor 1
CTL: Control group
LPS: Lipopolysaccharide
NO: Nitric oxide
iNOS: Inducible nitric oxide synthase
TNF-α: Tumor necrosis factor-alpha
IL: Interleukin
IFN-γ: Interferon
LC3: Microtubule-associated protein 1A/1B-light chain 3B
MAPKs: Mitogen-activated protein kinase
NF-kB: Nuclear factor kappa-light-chain-enhancer of activated B cells
FPKM: Fragments per kilobase of exon per million
BALF: Bronchoalveolar lavage fluid
Tg: Transgenic
GFP: Green fluorescent protein
