# Supplementary material for: Fludarabine attenuates inflammation and dysregulated autophagy in alveolar macrophages via inhibition of STAT1/IRF1 pathway
Source: Lab Anim Res. 2025 May 7;41:12. doi: 10.1186/s42826-025-00245-7 (PMC12057031; doi:10.1186/s42826-025-00245-7)
Supplement: Supplementary file 1 — Additional file 1. [file 42826_2025_245_MOESM1_ESM.docx]

**Supplementary Figure**


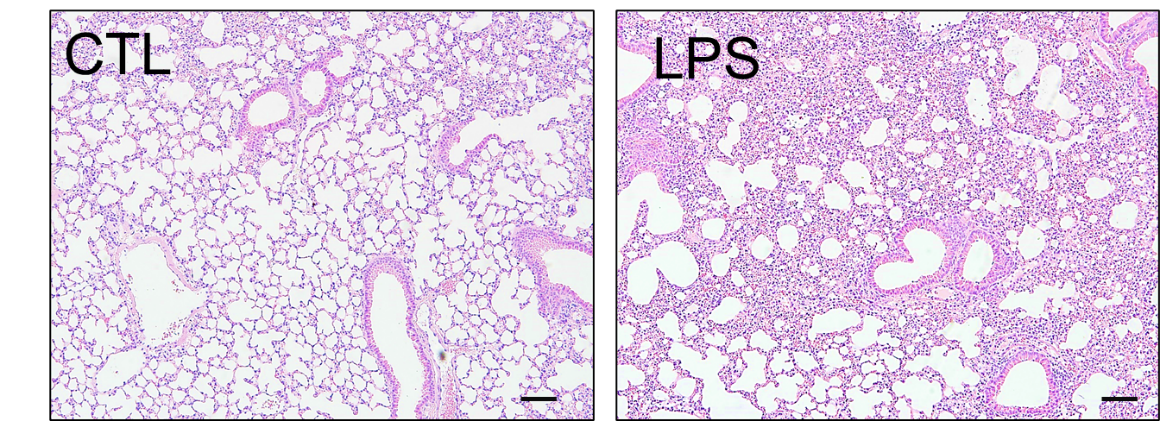


Fig S1. LPS induces acute lung injury in mice. CTL: control; LPS: lipopolysaccharide. Scale bar: 100 um.
